# CD36 rs1761667 Polymorphism and Its Impact on Molecular Signatures in Bladder Cancer

**DOI:** 10.3390/diseases14020044

**Published:** 2026-01-28

**Authors:** Mihai Ioan Pavalean, Ioana Maria Lambrescu, Gisela Gaina, Victor Lucian Madan, Mihail Eugen Hinescu, Laura Cristina Ceafalan

**Affiliations:** 1 Department of Morphological Sciences, Carol Davila University of Medicine and Pharmacy, 050474 Bucharest, Romania; mihai-ioan.pavalean@drd.umfcd.ro (M.I.P.); ioana.lambrescu@umfcd.ro (I.M.L.); victmad@gmail.com (V.L.M.); mhinescu@yahoo.com (M.E.H.); laura.ceafalan@umfcd.ro (L.C.C.); 2 Clinical Department 3, Dr. Carol Davila Central Military Emergency University Hospital, 010825 Bucharest, Romania; 3 Department of Cell Biology, Neuroscience and Experimental Myology, Victor Babes National Institute of Pathology, 050096 Bucharest, Romania; 4 Department of Pathology, Victor Babes National Institute of Pathology, 050096 Bucharest, Romania

**Keywords:** bladder cancer, *CD36* gene, rs1761667 polymorphism, tumor grade, inherited variation

## Abstract

Background: Bladder cancer remains a heterogeneous disease, and genetic factors are increasingly recognized as potential contributors to its pathogenesis. CD36, a multifunctional scavenger receptor implicated in lipid metabolism and tumor progression, has not been previously investigated in relation to bladder cancer-associated polymorphisms. Objectives: This study examined the relationship between the rs1761667 variant and CD36 mRNA expression. Methods: Our study included 30 patients with bladder cancer and 19 controls. PCR–RFLP genotyping for rs1761667 and RT–qPCR quantification of CD36 mRNA expression, with GAPDH as the reference gene, were performed. Expression levels were analyzed using the 2^−ΔΔCt^ method, and statistical significance was defined as *p* < 0.05. Results: In patients, CD36 expression varied significantly across rs1761667 genotypes with reduced expression in AA carriers compared with GG carriers (post hoc, *p* = 0.009, with a Holm-adjusted *p* = 0.03). No significant genotype-related differences were observed among controls. Genotype distributions did not differ significantly between cases and controls (χ^2^, *p* = 0.053). Conclusions: These results indicate that rs1761667 may modulate CD36 transcription in a genotype-dependent manner, particularly in the disease context. Overall, our findings point to a potential biological connection between inherited CD36 variation and bladder cancer-related pathways, underscoring the need for further validation in tumor tissues

## 1. Introduction

Bladder cancer (BC) is the tenth most common type of cancer worldwide and a major cause of both morbidity and mortality [1]. The World Health Organization reported 573,278 new BC diagnoses in 2020, and this number is expected to quadruple by 2040 [2,3]. Male incidence and mortality rates are 9.5 and 3.3 per 100,000, respectively, four times higher than those in women worldwide [2]. Based on cytological and architectural abnormalities, urothelial neoplasms are classified into low- and high-grade carcinomas [4]. Histopathologically, non-muscle-invasive bladder cancer (NMIBC) is confined to the urothelium and lamina propria (Ta/T1/Tis) [5], whereas muscle-invasive bladder cancer (MIBC) (T2–T4) is associated with a poor prognosis (5-year survival, 27–50%) [6]. Although invasive cystoscopy and biopsy are standard diagnostic approaches, their limited sensitivity may result in inaccurate assessment of tumor biology and suboptimal treatment decisions [6]. Therefore, for efficient, individualized treatment, a better understanding of the biochemical and metabolic variability of BC is necessary.

CD36, a class B scavenger receptor, has been shown to mediate fatty acid uptake and tumor progression [7,8]. Additionally, genomic, inflammatory, and metabolic mechanisms contribute to BC heterogeneity and progression, highlighting the importance of inherited genetic variation in tumor behavior [9]. CD36 has emerged as a poor prognostic factor in multiple malignancies, with immunohistochemical studies in BC demonstrating strong associations between CD36 expression and high-grade disease, lymph node invasion, increased tumor recurrence, and reduced survival [10].

The CD36 gene, located on chromosome 7q11.2 [11] and comprising 15 exons, harbors the common rs1761667 polymorphism, an intronic G > A variant in the 5′ flanking region of exon 1A [12]. Located in a 5′ flanking intronic region, rs1761667 may influence CD36 transcription through regulatory effects and has been reported as a putative blood eQTL, supporting a role in modulating CD36 mRNA expression at the systemic level.

CD36 polymorphisms, including rs1761667, have been extensively associated with cardiometabolic traits such as cardiovascular disease [13,14], type 2 diabetes [15], obesity [16], and fat intake or perception [17], and rs1761667 has been proposed as a prognostic marker in obese T2DM (type 2 diabetes mellitus) patients [18]. Beyond metabolic disease, rs1761667 has also been implicated in oncology, contributing to phenotypic variability in familial adenomatous polyposis and colorectal cancer risk [19,20], and has been associated with increased BC risk and elevated circulating CD36 levels in metastatic disease [21].

CD36 is highly expressed in circulating immune cells, where it contributes to lipid uptake, inflammatory signaling, and immune regulation. As systemic metabolic and inflammatory states can influence tumor behaviour and the tumor microenvironment, variation in CD36 expression in peripheral blood may therefore capture host-level mechanisms relevant to BC progression [22]. This provides a plausible biological rationale for analysing CD36 expression in blood as a complementary approach to tumor-based analyses.

Although there is increasing evidence that CD36 polymorphisms are associated with metabolic and oncological disorders, little is known about the connection between the rs1761667 (G > A) variant and CD36 mRNA expression in BC. Previous research on urothelial carcinoma has mostly examined CD36 protein expression in tumor tissue, ignoring the possibility that inherited CD36 variation contributes to inter-individual variation in gene expression. Given CD36′s predictive importance in numerous cancers, studying rs1761667′s functional effects in BC is important and potentially clinically relevant. Thus, our study investigated the CD36 rs1761667 polymorphism and CD36mRNA expression levels in BC patients, hypothesizing that germline variation may contribute to inter-patient heterogeneity relevant to disease progression and patient stratification.

## 2. Materials and Methods

### 2.1. Study Design/Participants/Inclusion Criteria/Clinical Evaluation

This case–control study included a total of 49 participants, comprising 30 patients diagnosed with BC and 19 healthy individuals serving as the control group. Patients were recruited from The Central Military Emergency University Hospital “Dr. Carol Davila” in Bucharest between December 2022 and December 2023 and were eligible for inclusion if they met the following criteria: (1) Histologically confirmed diagnosis of BC; (2) No prior treatment (chemotherapy, radiotherapy, or immunotherapy) at the time of sample collection; (3) Availability of complete clinical and demographic data; (4) Provided written informed consent for participation and blood sample collection. This research has received approval from the Research Ethics Committee of the Central Military Emergency University Hospital, Bucharest (Approval No. 556/20.12.2022), and written informed consent was obtained from all individuals in accordance with the Declaration of Helsinki of 1975 as revised in 2013. The control group consisted of age- and sex-matched healthy individuals with no history of malignancy or chronic inflammatory diseases. All participants provided peripheral blood samples, which were used for both genotyping and gene expression analysis. Genomic DNA was extracted to determine the CD36 rs1761667 polymorphism using the PCR-RFLP method (Polymerase Chain Reaction-Restriction Fragment Length Polymorphism). Total RNA was isolated for quantitative analysis of CD36 mRNA expression by real-time RT-PCR.

### 2.2. DNA Extraction and Genotyping

5 mL of peripheral blood from patients with BC were collected into EDTA anti-coagulation tubes. 2 mL were used for DNA isolation. The remaining portion of the blood samples was used for RNA isolation. Genomic DNA was extracted from leukocyte samples using a commercially available kit (PureLink™ Genomic DNA Mini Kit, Invitrogen, Carlsbad, CA, USA) according to the manufacturer’s instructions. The concentration and quality of the DNA extracts were assessed using a Nanodrop spectrophotometer (NanoDrop 2000, ThermoScientific, Waltham, MA, USA). The DNA samples were stored at −20 °C until use.

Genotypes of the 7q11.2 genomic region rs1761667 polymorphism in CD36 were detected using polymerase chain reaction-restriction fragment length polymorphism (PCR-RFLP). PCR amplifications were performed in a total volume of 15 μL of PCR Master Mix (Thermo Scientific™), using 50 ng of genomic DNA in the reaction and primers at a concentration of 0.09 μM each. Primer sequences used in this study are reported in Table 1. Primers amplify a fragment of 425 base pairs (bp). The PCR reaction was performed in a thermocycler (Mastercycler^®^ NexusX2, Eppendorf, Enfield, CT, USA) under the following conditions: initial denaturation at 95 °C for 5 min, followed by 35 cycles of amplification: denaturation at 95 °C, annealing at 57 °C, and extension at 72 °C (each comprising 30 s), and the final extension at 72 °C for 5 min. 10 μL of the PCR product, 2 μL of 10X Digest Buffer, 0.5 μL of BsuRI (HaeIII) ThermoScientific, USA, and 18 μL of nuclease-free water were used to digest the PCR products in a total volume of 20.5 μL. HaeIII digestion resulted in 264 bp and 161 bp fragments (G allele). The A allele generated one 425 bp fragment. Ethidium bromide staining was applied after restriction fragments were isolated using 2% agarose gel electrophoresis. The fragments were visualized under UV light using the UVP ChemiDoc-It Imaging System (Upland, CA, USA).

### 2.3. RNA Extraction and Gene Expression

Total RNA was extracted using a PureLink RNA Mini Kit (Invitrogen, ThermoScientific, Waltham, MA, USA) according to the manufacturer’s instructions. RNA integrity and purity were assessed using a NanoDrop 2000 (ThermoScientific, USA). Using the SuperScript IV VILO Master Mix (ThermoScientific, USA), 1 μg of RNA was reverse transcribed into cDNA under the following conditions: 10 min at 37 °C, followed by 10 min at 25 °C, 30 min at 50 °C, and 85 °C for 5 min after adding SuperScript Master Mix. cDNAs were stored at −20 °C until used. Quantitative real-time PCR (RT-qPCR) was performed using SYBR™ Green PCR Master Mix (Applied Biosystems, Foster City, CA, USA) in a QuantStudio™ 7 Flex Real-Time PCR System (Applied Biosystems, USA). Reactions were carried out in a final volume of 10 µL, containing 1 µL of 1:10 diluted cDNA and 0.3 µM of each primer (forward and reverse, see Table 1). The cycling conditions were as follows: 50 °C for 2 min, 95 °C for 10 min, followed by 40 cycles of 95 °C for 15 s and 60 °C for 1 min. Each sample was run in duplicate. GAPDH was used as the internal reference gene, and relative expression levels of CD36 were calculated using the 2^−ΔΔCt^ method [23], with the mean ΔCt value of the control group used as the calibrator. The use of GAPDH as a reference gene is supported by its expression stability, assessed across all blood samples and showing minimal variability with no significant differences between groups.

### 2.4. Statistical Analysis

Normality of continuous data was assessed with the Shapiro–Wilk test and variance homogeneity with Levene’s test. In patients, CD36 mRNA expression across rs1761667 genotypes was compared using the Kruskal–Wallis test; when significant, pairwise comparisons were examined with Dunn’s post hoc test and Holm correction. In controls, the Kruskal–Wallis test was used. Between-group comparisons (patients vs. controls) were performed using the Mann–Whitney U test or unpaired *t*-tests, as appropriate. Pearson’s χ^2^ compared categorical variables; Fisher’s exact test was applied when expected cell counts were <5. Linear trends across tumor grade were assessed with the Cochran–Armitage test. A *p* value < 0.05 was considered statistically significant. Hardy–Weinberg equilibrium was evaluated in the control group. SPSS version 26 (IBM Corp., Armonk, NY, USA). Given the pilot and exploratory nature of the study, the sample size was based on case availability rather than on a predefined power calculation. As such, no formal a priori power analysis was conducted.

## 3. Results

### 3.1. Demographic Study/Biochemical Characteristics

A total of 49 individuals were included: 30 patients with BC and 19 healthy controls. The two groups were matched for age and sex, with no significant differences in demographic variables (*p* > 0.05). The demographic and clinical characteristics of study participants are summarized in Table 2 and Table 3.

The mean age of BC patients (70.9 ± 10.57) was comparable to that of healthy controls (66.21 ± 9.89 years), indicating appropriate age matching between the groups. Within the case group, a clear male predominance was observed (76.7% males vs. 23.3% females), reflecting the known higher incidence of BC in men. As designed, the study cohort included a greater number of cases than controls to ensure sufficient statistical power for genetic and expression analyses.

We first assessed the distribution of rs1761667 genotypes and alleles in cases and controls. Subsequently, we compared CD36 mRNA expression levels between patients and healthy individuals and further explored the impact of rs1761667 genotypes on gene expression. Finally, correlations with clinicopathological characteristics of BC were evaluated to determine the clinical significance of these findings.

### 3.2. Genotypes and Allele Frequencies

**Figure 1:**
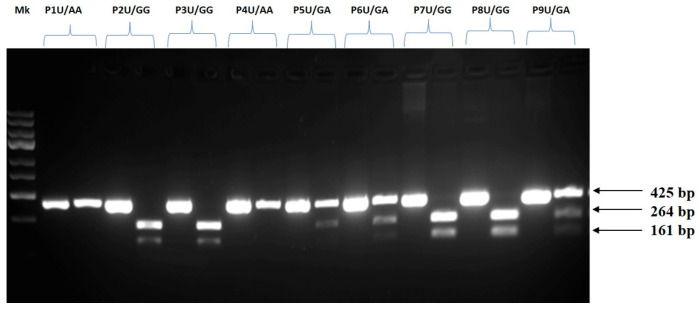
PCR-RFLP genotyping of the CD36 rs1761667 locus. Representative 2% agarose gel electrophoresis of PCR-RFLP products for the CD36 rs1761667 (7q11) locus, showing undigested (U) and digested amplicons. The three genotypes are illustrated as follows: AA—a single band at 425 bp; GG—two bands at 264 bp and 161 bp; GA—three bands at 425 bp, 264 bp, and 161 bp. Mk = DNA ladder (O’GeneRuler Express DNA Ladder, Thermo Scientific, USA); P = patient sample; U = undigested sample. The original, uncropped gel is presented in Supplementary Figure S1.

PCR-RFLP analysis confirmed the presence of three rs1761667 genotypes (GG, GA, and AA) in the study population. Figure 1 illustrates the successful PCR amplification, showing the three different genotypes.

**Figure 2:**
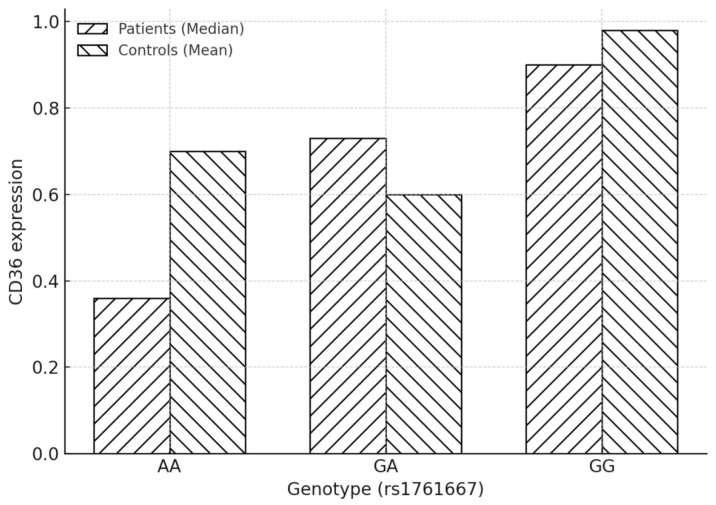
Graphical representation of the CD36 expression in patients and controls by genotype. Bars represent the median CD36 mRNA expression values for each genotype (AA, GA, GG), as determined by qPCR analysis. Hatched bars indicate median expression levels in patients, while cross-hatched bars indicate controls. In patients, CD36 expression showed a gradual increase from AA (0.36) to GA (0.73) and GG (0.90). In contrast, the median expression in controls was highest in the GG group (0.97), followed by AA (0.71) and GA (0.46).

Table 4 and Table 5, together with Figure 2, summarize the distribution of rs1761667 genotypes and CD36 expression levels in patients and controls, with the genotype distribution specifically detailed in Table 3. Among patients, genotype frequencies were GG = 33.3%, GA = 50.0%, and AA = 16.7%, whereas in controls, GG = 57.9%, GA = 15.8%, and AA = 26.3%. Statistical analysis revealed no significant difference between groups (χ^2^ test, *p* = 0.053). Genotype frequencies in controls deviated from Hardy–Weinberg equilibrium (χ^2^ = 8.01, *p* = 0.018), most likely due to the modest sample size.

### 3.3. qPCR Analysis/CD36 mRNA Expression in Patients with BC

CD36 expression at the mRNA levels was assessed by real-time PCR after genotyping participants for the rs1761667 polymorphism (GG, GA, and AA). Table 6 summarizes CD36 expression levels in patients and controls according to rs1761667 genotypes. In patients, CD36 expression values were reported as median (IQR) due to non-normal distribution. For the controls, in the context of genotype-specific analysis, the distribution was found to be non-parametric; therefore, we applied the Kruskal–Wallis test. The median expression values in patients increased progressively from AA (0.36) to GA (0.73) and GG (0.90). In contrast, the median expression levels in the controls were highest in the GG group (0.97), followed by the AA group (0.71) and the GA group (0.46).

In patients with BC, Kruskal–Wallis analysis demonstrated significant differences in CD36 expression across rs1761667 genotypes (H(2) = 7.84, *p* = 0.02). Post hoc pairwise comparisons indicated that CD36 expression was significantly higher in GG compared with AA carriers (post hoc *p* = 0.009, with a Holm-adjusted *p* = 0.03). At the same time, GA did not differ significantly from either AA (*p* = 0.073) or GG (*p* = 0.114). Given the limited number of individuals within each genotypic group, especially AA carriers, analyses stratified by genotypes should be considered exploratory.

Regarding genotype distribution, it did not differ significantly between cases and controls—Pearson test χ^2^ (χ^2^(2) = 5.87, *p* = 0.053) (Table 6).

In the control group, Kruskal–Wallis analysis showed no significant differences in CD36 expression across genotypes (H(2) = 3.13, *p* = 0.209).

By enabling the uptake of long-chain fatty acids, CD36 participates in lipid signaling pathways and influences both lipid storage and oxidative metabolism in diverse tissues. In this context and given the availability of patient data documenting the presence or absence of dyslipidemia, we evaluated these two variables within our cohort.

The distribution of rs1761667 genotypes according to dyslipidemia status is shown in Table 7. Among patients without dyslipidemia, the proportions of AA, GG, and GA carriers were 80.0%, 90.0%, and 66.7%, respectively, compared with 20.0%, 10.0%, and 33.3% among those with dyslipidemia. Chi-square analysis did not reveal a statistically significant association between genotype and dyslipidemia (Pearson χ^2^(2) = 1.86, *p* = 0.394).

As presented in Table 8, no deaths occurred within two years among patients with grade 1 or grade 2 tumors, while 50% of those with grade 3 tumors had died. Chi-square analysis indicated a significant association between tumor grade and 2-year mortality (Pearson χ^2^(2) = 7.50, *p* = 0.024). Moreover, linear-by-linear association analysis demonstrated a significant trend (*p* = 0.013), indicating that higher tumor grade was associated with an increased risk of death within two years.

## 4. Discussion

In this study, we investigated the relationship between the CD36 rs1761667 polymorphism and CD36 mRNA expression levels in BC. Importantly, no statistically significant association between rs1761667 genotype distribution and BC risk was observed in this study. Although the rs1761667-expression relationship has been explored in cardiometabolic contexts (12), as well as in athlete injury risk [19], to our knowledge, no prior study has directly evaluated this variant in relation to CD36 mRNA levels in BC. The present data, therefore, extend the functional relevance of rs1761667 to urothelial malignancy. No significant difference in genotype frequencies was observed between cases and controls (*p* = 0.053); this finding should be interpreted with caution, given the sample size. However, the within-patient genotype–expression gradient supports the biological plausibility of a genotype-dependent effect, while remaining exploratory.

The key findings of our investigation can be summarized as follows, providing insights into the functional role of rs1761667 in BC.

Genotype and CD36 mRNA level. In this case–control study, we observed a genotype–expression association whereby rs1761667 was associated with CD36 mRNA levels in patients with BC (AA < GA < GG), with a significant global difference across genotypes (Kruskal–Wallis *p* = 0.02) driven by lower expression in AA versus GG (post hoc *p* = 0.009). In contrast, no significant differences were observed among the controls (Kruskal–Wallis H(2) = 3.13, *p* = 0.209). Taken together, these findings are consistent with an association between rs1761667 genotype and variability in CD36 mRNA expression, with the effect observed specifically in the disease context. Clinically, CD36 sits at the nexus of fatty-acid uptake, inflammation, and tumor–stroma crosstalk; thus, genotype-linked variation in expression may warrant further investigation into metabolic heterogeneity with potential implications for risk stratification and therapeutic vulnerability (e.g., pathways tied to lipid uptake/oxidation). Higher CD36 activity can support metabolic plasticity, invasion, and adverse outcomes across various malignancies, including urothelial cancer. The observed genotype-dependent gradient in CD36 expression (AA → GG) in BC patients aligns with a model in which rs1761667 influences transcriptional output, while disease-specific cues (tumor microenvironment, systemic metabolic/inflammatory milieu) amplify this effect in patients but not in healthy controls. Taken together, our results position rs1761667 as a putative functional determinant of CD36 expression in BC, with potential downstream impact on tumor metabolic programming.

CD36 and dyslipidemia. In our cohort, the rs1761667 variant showed no significant association with dyslipidemia (Pearson χ^2^, *p* = 0.394). The lack of association between CD36 and dyslipidemia should be interpreted cautiously. Given the modest sample size and the potential influence of medication use, adiposity, insulin resistance, and hepatic steatosis, these factors cannot be excluded. Future studies in larger, well-phenotyped cohorts—using fasting lipid panels, apolipoproteins, insulin resistance indices, liver fat quantification, and detailed medication logs—are needed to delineate the relationship between CD36 and systemic lipid metabolism.

CD36 and smoking. Although smoking is a major risk factor for bladder carcinogenesis and can alter systemic inflammation and lipid handling, we did not observe a clear pattern linking rs1761667 genotype or CD36 mRNA with smoking status in this cohort, with the limited sample size reducing the power to detect subtle genotype- or expression-related effects.

Tumor grade and 2-year mortality. In our cohort, 2-year mortality increased across histological grades, being 0% in G1, 0% in G2, and 50% in G3 (10/20 deaths). This association was statistically significant (Pearson χ^2^(2) = 7.50, *p* = 0.024) with evidence of a dose–response relationship indicating an increasing risk with higher grade (*p* = 0.013). These data reinforce the prognostic relevance of histologic grade and are biologically plausible given the greater proliferative and invasive potential of high-grade tumors. Considered alongside other known prognostic factors in BC, the results are consistent with current knowledge and show no direct association with CD36 genotype or expression.

Several limitations should be acknowledged. The study was conducted without an a priori power calculation and with relatively small sample sizes, resulting in limited statistical power, particularly for subgroup analyses. The small sample size may also account for the deviation from Hardy–Weinberg equilibrium observed in the control group. While peripheral blood CD36 mRNA expression may not fully reflect tumor-level expression, it provides a complementary, exploratory host-level perspective alongside tumor-focused studies. Our findings should be validated in larger, independent cohorts, ideally incorporating tumor tissue expression data and long-term clinical outcomes. In addition, metabolomic and lipidomic profiling combined with CD36 genotyping may help elucidate the metabolic pathways linking lipid handling and metabolic plasticity to BC development.

## 5. Conclusions

In summary, although genotype and allele frequencies did not differ significantly between patients and controls, CD36 mRNA levels displayed a clear genotype-dependent trend in BC cases, with lower expression in AA carriers. These findings support the hypothesis that rs1761667 may influence CD36 transcription in a disease-specific context. Given the small sample size, deviation from Hardy–Weinberg equilibrium in controls, and the use of peripheral blood rather than tumor tissue, these results should be considered preliminary. Larger studies with tumor-based analyses are required to confirm the functional relevance of rs1761667, clarify its potential value as a biomarker of metabolic heterogeneity in BC, and further explore its association with metabolic heterogeneity in BC.

## Figures and Tables

**Table 1:** Primer sequences used in this study.

Target	Application	Primer Sequence (5′ → 3′)
rs1761667	PCR-RFLP	F: CAAGGTCTGGTATCCACCTGTT R: ATGAAGCTTCCCGCCTTAGAA
CD36	RT-qPCR	F: GGCTGTGACCGGAACTGTG R: AGGTCTCCAACTGGCATTAG
GAPDH	RT-qPCR	F: ACCCACTCCTCCACCTTTGA R: CTGTTGCTGTAGCCAAATTCG

**Table 2:** Demographic and clinical characteristics of study participants.

	Total (*n* = 30)			NMIBC (*n* = 25, 83.3%)			MIBC (*n* = 5, 16.7%)		
**Sex**	F (*n* = 7, 23.3%)			F (*n* = 4, 16%)			F (*n* = 3, 60%)		
	M (*n* = 23, 76.7%)			M (*n* = 21, 84%)			M (*n* = 2, 40%)		
	**Mean ± SD**		**Min–max**	**Mean ± SD**		**Min–max**	**Mean ± SD**		**Min** **–max**
**Age**	70.9 ± 10.57		39–85	70.32 ± 11.13		39–85	73.80 ± 7.29		64–83
**BMI**	28.51 ± 7.02		20.76–55.1	29.41 ± 7.32		20.76–55.1	24.01 ± 2.36		20.76–27.18
	**Yes (*n*, %)**	**No (*n*, %)**		**Yes (*n*, %)**	**No (*n*, %)**		**Yes (*n*, %)**	**No (*n*, %)**	
**Smoker (available for 27p)**	14 (51.9)	13 (48.1)		12 (57.1)	9 (42.9)		2 (33.3)	4 (66.7)	
**Obesity**	8 (25.8)	23 (74.2)		7 (28)	18 (72)		1 (16.7)	5 (83.3)	
**Hypertension (28p)**	11 (39.3)	17 (60.7)		10 (45.5)	12 (54.5)		1 (16.7)	5 (83.3)	
**Diabetes**	4 (12.9)	27 (87.1)		4 (16)	21 (84)		0	6 (100)	
**Heart failure (30p)**	4 (13.3)	26 (86.7)		3 (12.5)	21 (87.5)		1 (16.7)	5 (83.3)	
**Coronary heart disease (30p)**	1 (3.3)	29 (96.7)		1 (4.2)	23 (95.8)		0	6 (100)	
**Dyslipidemia (30p)**	7 (23.3)	23 (76.7)		7 (28)	18 (72)		0	5 (100)	

**Table 3:** Control group demographics.

*n* = 19 (%)		
Age	66.21 ± 9.89% (mean, SD)	45–84 (min–max)
Sex	F: 2 (10.5%), M: 17 (89.5%)	—

**Table 4:** Distribution of CD36 rs1761667 genotypes in patients and controls.

Genotypic Frequencies	Patients		Control		*p* Value
	Total (*n* = 30)	(%)	Total (*n* = 19)	(%)	
AA	5	16.7%	5	26.3%	
GA	15	50%	3	15.8%	0.053
GG	10	33.3%	11	57.9%	

**Table 5:** Mean and median values of CD36 expression in the patient and control groups.

Genotype	Patients *n* = 30 (%)	Controls *n* = 19 (%)	CD36 Expression (Patients) Median (IQR)	CD36 Expression (Controls) Median (IQR)
AA	5 (16.7%)	5 (26.3%)	0.36 (0.60)	0.71 (0.30)
GA	15 (50.0%)	3 (15.8%)	0.73 (0.63)	0.46 (0.31)
GG	10 (33.3%)	11 (57.9%)	0.90 (0.73)	0.97 (0.21)

**Table 6:** CD36 mRNA expression across rs1761667 genotypes in patients with BC and controls.

Genotype	Patients, Median (IQR)	Controls, Median (IQR)
AA	0.36 (0.60)	0.71 (0.30)
GA	0.73 (0.63)	0.46 (0.31)
GG	0.90 (0.73)	0.97 (0.21)

In patients, CD36 expression differed significantly across rs1761667 genotypes (Kruskal–Wallis H(2) = 7.84, *p* = 0.02); post hoc analysis showed higher expression in GG vs. AA (*p* = 0.009), while GA did not differ significantly from AA or GG. In controls, no significant genotype-dependent differences were observed (Kruskal–Wallis H(2) = 3.13, *p* = 0.209). Genotype distribution did not differ significantly between patients and controls (Pearson χ^2^ = 5.87, *p* = 0.053).

**Table 7:** Distribution of rs1761667 genotypes according to dyslipidemia (patients, *n* = 30).

**Genotype**	**No Dyslipidemia (*n*, %)**	**Dyslipidemia (*n*, %)**	**Total**	Pearson χ^2^ = 1.863, df = 2, *p* = 0.394
AA	4 (80.0%)	1 (20.0%)	5	
GG	9 (90.0%)	1 (10.0%)	10	
GA	10 (66.7%)	5 (33.3%)	15	
Total	23 (76.7%)	7 (23.3%)	30	

**Table 8:** Association between tumor grade and 2-year mortality (*n* = 30).

Tumor Grade	Alive at 2 Years *n* (%)	Death at 2 Years *n* (%)	Total *n* (%)
G1	3 (100%)	0	3 (10%)
G2	7 (100%)	0	7 (23.3%)
G3	10 (50%)	10 (50%)	20 (66.7%)
Total	20 (66.7%)	10 (33.3%)	30 (100%)

## Data Availability

The raw data supporting the conclusions of this article will be made available by the authors, without undue reservation.

## Supplementary Materials

The following supporting information can be downloaded at: https://www.mdpi.com/article/10.3390/diseases14020044/s1, Figure S1: The original, uncropped gel images.

## Abbreviations

The following abbreviations are used in this manuscript:

BC	Bladder cancer
bp	Base pair
IQR	Interquartile range
MIBC	Muscle-invasive bladder cancer
NMIBC	Non-muscle-invasive bladder cancer
PCR-RFLP	Polymerase chain reaction–restriction fragment length polymorphism
RT-qPCR	Reverse transcription quantitative PCR
T2DM	Type 2 diabetes mellitus
SD	Standard deviation

## References

[1] Lobo N., Afferi L., Moschini M., Mostafid H., Porten S., Psutka S.P., Gupta S., Smith A.B., Williams S.B., Lotan Y. (2022). Epidemiology, Screening, and Prevention of Bladder Cancer. Eur. Urol. Oncol..

[2] Sung H., Ferlay J., Siegel R.L., Laversanne M., Soerjomataram I., Jemal A., Bray F. (2021). Global Cancer Statistics 2020: GLOBOCAN Estimates of Incidence and Mortality Worldwide for 36 Cancers in 185 Countries. CA Cancer J. Clin..

[3] Antoni S., Ferlay J., Soerjomataram I., Znaor A., Jemal A., Bray F. (2017). Bladder Cancer Incidence and Mortality: A Global Overview and Recent Trends. Eur. Urol..

[4] Raspollini M.R., Comperat E.M., Lopez-Beltran A., Montironi R., Cimadamore A., Tsuzuki T., Netto G.J. (2023). News in the classification of WHO 2022 bladder tumors. Pathologica.

[5] Matuszczak M., Kiljańczyk A., Salagierski M. (2022). A Liquid Biopsy in Bladder Cancer—The Current Landscape in Urinary Biomarkers. Int. J. Mol. Sci..

[6] Witjes J.A., Bruins H.M., Cathomas R., Compérat E.M., Cowan N.C., Gakis G., Hernández V., Espinós E.L., Lorch A., Neuzillet Y. (2021). European Association of Urology Guidelines on Muscle-invasive and Metastatic Bladder Cancer: Summary of the 2020 Guidelines. Eur. Urol..

[7] Wang J., Li Y. (2019). CD36 tango in cancer: Signaling pathways and functions. Theranostics.

[8] Pardo J.C., Sanhueza T., de Porras V.R., Etxaniz O., Rodriguez H., Martinez-Cardús A., Grande E., Castellano D., Climent M.A., Lobato T. (2022). Prognostic Impact of CD36 Immunohistochemical Expression in Patients with Muscle-Invasive Bladder Cancer Treated with Cystectomy and Adjuvant Chemotherapy. J. Clin. Med..

[9] Bizzarri F.P., Campetella M., Russo P., Palermo G., Moosavi S.K., Rossi F., D’amico L., Cretì A., Gavi F., Panio E. (2025). Prognostic Value of PLR, SIRI, PIV, SII, and NLR in Non-Muscle Invasive Bladder Cancer: Can Inflammatory Factors Influence Pathogenesis and Outcomes?. Cancers.

[10] El Maged A.M.A., Badr N.M., Mohamed H.L. (2025). An Insight into Prognostic Impact of TIPE2 & CD36 Immunohistochemical Expression in Urothelial Carcinoma. Iran. J. Pathol..

[11] Rać M.E., Safranow K., Poncyljusz W. (2007). Molecular Basis of Human CD36 Gene Mutations. Mol. Med..

[12] Yazdanpanah Z., Salehi-Abargouei A., Mollahosseini M., Sheikhha M.H., Mirzaei M., Mozaffari-Khosravi H. (2023). The cluster of differentiation 36 (CD36) rs1761667 polymorphism interacts with dietary patterns to affect cardiometabolic risk factors and metabolic syndrome risk in apparently healthy individuals. Br. J. Nutr..

[13] Zhang Y., Ling Z., Deng S., Du H., Yin Y., Yuan J., She Q., Chen Y. (2014). Associations between CD36 gene polymorphisms and susceptibility to coronary artery heart disease. Braz. J. Med. Biol. Res..

[14] Boghdady A., Arafa U.A., Sabet E.A., Salama E., El Sharawy A., Elbadry M.I. (2016). Association between rs1761667 polymorphism of CD36 gene and risk of coronary atherosclerosis in Egyptian population. Cardiovasc. Diagn. Ther..

[15] Banerjee M., Gautam S., Saxena M., Bid H.K., Agrawal C. (2010). Association of CD36 gene variants rs1761667 (G>A) and rs1527483 (C>T) with Type 2 diabetes in North Indian population. Int. J. Diabetes Mellit..

[16] Sayed A., Šerý O., Plesnik J., Daoudi H., Rouabah A., Rouabah L., A Khan N. (2015). CD36 AA genotype is associated with decreased lipid taste perception in young obese, but not lean, children. Int. J. Obes..

[17] Bayoumy N.M., El-Shabrawi M., Hassan H.H. (2012). Association of cluster of differentiation 36 gene variant rs1761667 (G > A) with metabolic syndrome in Egyptian adults. Saudi Med. J..

[18] Shukla A.K., Shamsad A., Kushwah A.S., Singh S., Usman K., Banerjee M. (2024). CD36 gene variant rs1761667(G/A) as a biomarker in obese type 2 diabetes mellitus cases. Egypt. J. Med. Hum. Genet..

[19] Holmes M., Connor T., Oldmeadow C., Pockney P.G., Scott R.J., Talseth-Palmer B.A. (2018). CD36—A plausible modifier of disease phenotype in familial adenomatous polyposis. Hered. Cancer Clin. Pract..

[20] Abdel-Hamed A.R., Fakhry M.M., Mesbah N.M., Abo-Elmatty D.M., Sayed-Ahmed M.M., Osman A.-M.M., Ahmed O.S. (2024). CD-36 variants and circulating miRNAs as prognostic biomarkers and potential therapeutic targets in breast cancer patients. Gene Rep..

[21] El Ouali E.M., Kartibou J., Del Coso J., El Makhzen B., Bouguenouch L., El Akbir R., El Haboussi A., Akhouayri O., Ibrahimi A., Mesfioui A. (2025). Exploring the Association Between CD36 rs1761667 Polymorphism and Susceptibility to Non-Contact Tissue Injuries in Moroccan Elite Cyclists and Field Hockey Players: A Pilot Study. Genes.

[22] Alattar A.G., Storry J.R., Olsson M.L. (2024). Evidence that CD36 is expressed on red blood cells and constitutes a novel blood group system of clinical importance. Vox Sang..

[23] Livak K.J., Schmittgen T.D. (2001). Analysis of relative gene expression data using real-time quantitative PCR and the 2−ΔΔCT Method. Methods.

