# Supplementary figures and images for: CD36 rs1761667 Polymorphism and Its Impact on Molecular Signatures in Bladder Cancer
Source: Diseases. 2026 Jan 28;14(2):44. doi: 10.3390/diseases14020044 (PMC12938890; doi:10.3390/diseases14020044)

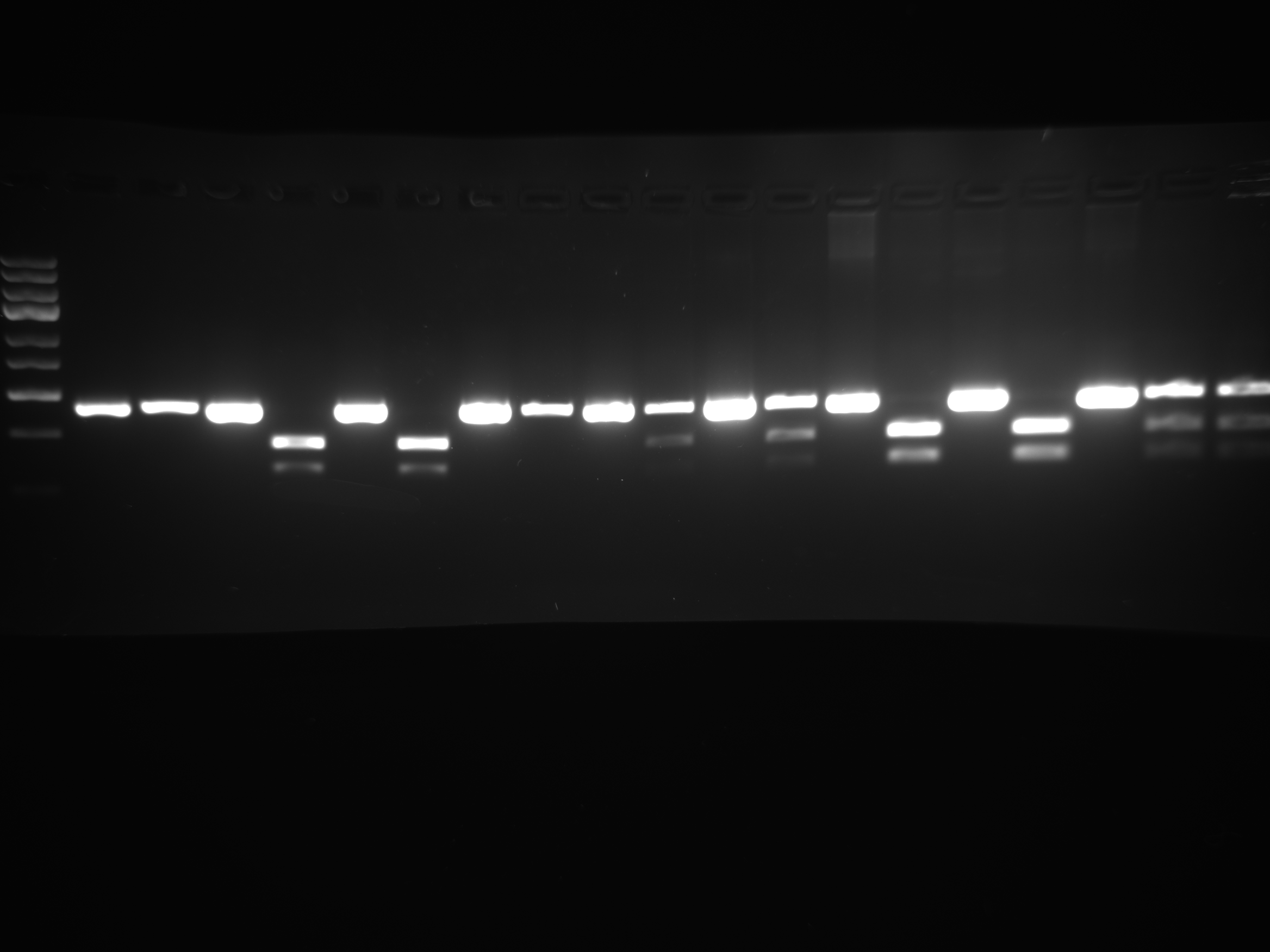

Supplement: Supplementary file 1 [file diseases-14-00044-s001.zip › diseases-4028338-supplementary.TIF]
